# Cancer stemness in hepatocellular carcinoma: mechanisms and translational potential

**DOI:** 10.1038/s41416-020-0823-9

**Published:** 2020-03-31

**Authors:** Yu-Man Tsui, Lo-Kong Chan, Irene Oi-Lin Ng

**Affiliations:** 1 Department of Pathology, The University of Hong Kong, Hong Kong, Hong Kong; 2 State Key Laboratory of Liver Research, The University of Hong Kong, Hong Kong, Hong Kong

**Keywords:** Hepatocellular carcinoma, Cancer stem cells

## Abstract

Cancer stemness, referring to the stem-cell-like phenotype of cancer cells, has been recognised to play important roles in different aspects of hepatocarcinogenesis. A number of well-established cell-surface markers already exist for liver cancer stem cells, with potential new markers of liver cancer stem cells being identified. Both genetic and epigenetic factors that affect various signalling pathways are known to contribute to cancer stemness. In addition, the tumour microenvironment—both physical and cellular—is known to play an important role in regulating cancer stemness, and the potential interaction between cancer stem cells and their microenvironment has provided insight into the regulation of the tumour-initiating ability as well as the cellular plasticity of liver CSCs. Potential specific therapeutic targeting of liver cancer stemness is also discussed. With increased knowledge, effective druggable targets might be identified, with the aim of improving treatment outcome by reducing chemoresistance.

## Background

Liver cancer is one of the most aggressive malignancies, with a high rate of recurrence in part due to the existence of cancer stem cells (CSCs), which contribute to resistance to chemotherapy. CSCs are cells that, like normal stem cells, have the ability to self-renew by dividing as well as to give rise to all cell types within a tumour; consequently, they are also referred to as tumour-initiating cells (TICs).^1–4^ The strong emphasis on tumour initiation makes the in vivo tumorigenicity assay a gold standard for defining cancer stemness. Therefore, in this review, only those studies with validation in tumorigenicity in vivo will be included for the discussion regarding cancer stemness in hepatocellular carcinoma (HCC).

Liver CSCs (LCSCs) are regulated by the expression of multiple stemness-related genes, such as Oct4, Sox2, Nanog and SALL4. For example, Oct4 and Sox2 are two transcription factors that are required for the self-renewal and pluripotency of embryonic stem cells.^5^ SALL4 is a transcription factor enhanced in epithelial cell adhesion molecule (EpCAM)^+^ AFP^+^ hepatic stem-cell-like (HpSC) subtype as compared with the mature hepatocyte (MH) subtype.^6^ In addition, Nanog is the downstream mediator of the signal transducer and activator of transcription 3 (STAT3) signalling pathway in CD24-promoted cancer stemness in HCC.^7^ LCSCs within HCC are mostly marked by various surface markers, some of which, such as CD13,^8^ CD24,^7,9^ CD44,^9^ CD133^9–11^ and EpCAM,^12,13^ are well established, although newer markers of LCSCs have also been identified. Genetic and epigenetic elements are known to affect various signalling pathways and to contribute to regulating cancer stemness. Furthermore, the tumour microenvironment is known to play an important role in regulating cancer stemness, and the potential interaction between CSCs and their microenvironment has provided insight into the cellular plasticity of LCSCs. Given the importance of LCSCs in mediating chemoresistance, potential ways of specific therapeutic targeting of liver cancer stemness have also been proposed.

## Markers for cancer stemness in the liver

Below we will briefly describe some well-established markers for cancer stemness in HCC, as well as discussing some representative potential new markers (Table 1).

**Table 1:** Summary of HCC cancer stemness markers that have been validated by tumorigenicity assays.

CSC markers	Functions in liver cancer stem cells	Subcellular localisation	References
Well-established markers			
CD13	Enhances side population, sphere formation, tumorigenicity, 5-FU and doxorubicin resistance; protects from ROS-induced DNA damage	Cell surface	^14,15^
CD24	Promotes cisplatin resistance, sphere formation, stemness gene expression, migration and invasion, tumorigenicity and STAT3 signalling to enhance Nanog expression; co-expression with CD133 promotes secondary tumour formation in vivo by inducing iNOS and Notch signalling	Cell surface	^7,16^
CD44	Promotes sorafenib resistance, sphere formation and tumorigenicity; helps maintain c-Met-induced stemness phenotypes	Cell surface	^17,18^
CD133	Promotes sphere formation, colony formation, stemness gene expression and tumorigenicity	Cell surface	^10^
EpCAM	Promotes sphere formation, invasion, 5-FU resistance, tumorigenicity and Wnt/β-catenin signalling; promotes EpCAM^+^ AFP^+^ hepatic stem-cell-like (HpSC) subtype; enhances SALL4 expression	Cell surface	^6,12^
Potential new markers			
ICAM-1	Promotes sphere formation, tumorigenicity and lung metastases; forms a feed-forward loop with Nanog	Cell surface	^26^
LGR5	Promotes stemness gene expression, sphere formation, tumorigenicity, and sorafenib and cisplatin resistance; stimulates hepatocyte and bile duct regeneration upon liver damage; enhances Wnt/β-catenin signalling	Cell surface	^27,29,31,32^
MAEL	Promotes stemness gene expression, EMT, migration and invasion, cisplatin resistance and tumorigenicity	Cytoplasmic	^29,31,32,36^
Cripto-1	Promotes stemness gene expression, migration and invasion, sphere formation, sorafenib and cisplatin resistance and tumorigenicity; stabilises Dvl3 protein to promote Wnt/β-catenin signalling	Cytoplasmic	^35,36^

### Well-established stemness markers

As demonstrated by enhanced tumorigenicity shown by the CD13^+^ fraction of HCC cells, CD13 is a marker of cancer stemness in dormant HCC cells, which are slowly cycling with resistance to chemotherapy/radiation therapy, as well as having the capability to reconstitute tumours.^14^ Only CD13^+^CD166^−^ cells could propagate to generate all of the original CD13^+^CD166^+^, CD13^+^CD166^−^, CD13^−^CD166^+^ and CD13^−^CD166^−^ subpopulations, while CD13^−^CD166^+^ cells could not,^15^ indicating that the CD13^+^CD166^−^ subpopulation could maintain the cancer stemness feature in a hierarchical manner, in which only the CSCs subset of HCC cells is able to self-renew by asymmetric and symmetric divisions to derive one and two daughter cells, respectively, capable of self-renewal and multipotency to prolong the continuous clonal growth of tumours.^3^

CD24 expression was found to be enriched in cisplatin-resistant HCC cells,^7^ and CD24^+^ HCC cell lines and patient-derived CD24^+^ HCC cells have enhanced sphere formation, expression of stemness genes and tumorigenicity compared with their CD24^−^ counterparts.^7^ Further mechanistic studies showed that the STAT3/Nanog signalling axis mediated these cancer stemness phenotypes.^7^ In another study, CD24^+^CD133^+^ HCC cells, but not CD24^−^CD133^+^ HCC cells, could form liver tumours after secondary orthotopic transplantation, and this process was mediated by signalling through inducible nitric oxide synthase (iNOS), tumour necrosis factor (TNF)-converting enzyme (TACE; also known as a disintegrin and metalloprotease domain [ADAM17]) and Notch.^16^

CD44^+^ cells demonstrated enhanced sphere-forming ability and tumorigenicity and were found in sorafenib-resistant HCC cell lines,^17^ whereas knockdown of CD44 could suppress HCC tumorigenicity.^18^ The co-expression of c-Met and CD44 in CD44^+^ HCC cells facilitated the maintenance of c-Met-induced stemness phenotypes.^18^ Furthermore, CD44^+^CD133^+^ cells had enhanced sphere-forming ability, and this population of cells was induced by maternal embryonic leucine zipper kinase, which was overexpressed in HCC samples and associated with poorer recurrence-free survival.^19^

CD133^+^ HCC cells had enhanced expression of stemness genes, sphere formation and tumorigenicity.^10^ Toll-like receptor 4 (TLR4)/Akt signalling was shown to promote cancer stemness in HCC cell lines, as reflected by an increase in the CD133^+^CD49^+^ HCC cell population, by upregulating Sox2.^20^ The enhanced tumorigenicity of CD133^+^CD49^+^ HCC cells was also Nanog dependent,^21^ as CD133^+^Nanog^high^ cells had increased expression of insulin-like growth factor 2 (IGF2) mRNA-binding protein-3 and YAP1, which ultimately inhibited transforming growth factor-β (TGF-β) signalling from suppressing TLR4 signalling.^21^

EpCAM is a well-established cancer stemness surface marker in HCC and can be induced by Wnt/β-catenin signalling.^12,22–25^ A specific hepatic stem cell-like subtype of EpCAM^+^ cells, but not an MH subtype of HCCs, was found to overexpress SALL4, a transcription factor that is known to regulate stemness in embryonic and haematopoietic stem cells, and further functional studies indicated a role for SALL4 in promoting cancer stemness in HCC.^6^

### Potential new markers of stemness

Cells that express intercellular adhesion molecule 1 (ICAM-1^+^ cells), sorted from HCC cell lines and human primary HCC tissues, had higher tumorigenicity than their ICAM-1^−^ counterparts.^26^ Conversely, liver-specific knockdown of ICAM-1 in hepatitis B virus (HBV) transgenic mice led to a reduction in the formation of liver tumours, as well as liver and lung metastases.^26^ Further investigation showed that ICAM-1 was transcriptionally regulated by Nanog.^26^

Interleukin-1 receptor-associated kinase 1 was reported to be overexpressed in HCC,^27^ and its knockdown in HCC cells suppressed sphere formation, tumorigenicity and protein expression of CD24 and CD47, which were enriched in TICs to promote cancer stemness features such as sphere formation and tumorigenicity,^28^ and chemoresistance to doxorubicin and sorafenib.^27^

Damage-induced leucine-rich repeat containing G-protein-coupled receptor 5 (LGR5)^+^ cells have been shown to regenerate hepatocytes and bile ducts in vivo,^29^ and LGR5 has pro-survival and pro-cancer stemness roles in HCC cells.^30,31^ In the presence of R-spondin, LGR5 activates Wnt/β-catenin signalling and functions downstream of the Dishevelled 3 (Dvl3)-mediated homeodomain-interacting protein kinase 2/catalytic subunit of protein phosphatase 1α/itchy E3 ubiquitin protein ligase homologue axis to promote cancer stemness phenotypes.^32^ Although knockdown of LGR5 suppressed tumorigenicity,^32^ its overexpression has not been consistently observed across HCC cohorts in different studies,^31–33^ so whether LGR5 is a genuine marker of cancer stemness in HCC needs further study.

The IGF signalling pathway is also implicated in cancer stemness of HCC as a high ratio of the insulin receptor isoform A (IR-A) to isoform B (IR-B) was associated with a higher expression of the hepatic progenitor cell (HPC) marker, CK19, and poorer overall survival,^34^ and there was enhanced IR-A expression in spheroid cultures. The ligands of IR, insulin and IGF2, induced the phosphorylation of IR and Akt in HCC cells, and stable overexpression of IR-A in an HCC cell line promoted migration and invasion, tumour growth, tumorigenicity and expression of stemness markers (CD133, CD44).^34^ These indicate that IR-A is a functional surface molecule to promote cancer stemness of HCC.

Cripto-1, a member of the epidermal growth factor-CFC family of proteins, is a protein overexpressed in HCC and associated with poorer overall and disease-free survival as it conferred stemness phenotypes in HCC cells by binding to Dvl3 protein and maintaining its stability,^35^ which promoted Wnt/β-catenin signalling.^32^

Not all markers of cancer stemness are only expressed on the cell surface. For example, Maelstrom protein, which is localised in the perinuclear structure and nucleus, is overexpressed in HCC and promotes many stemness phenotypes, including sphere formation and tumorigenicity in HCC cells.^36^

## Genetic elements that are relevant to liver cancer stemness

The HCC mutational landscape is characterised by pronounced intertumoural heterogeneity and the lack of a representative actionable oncogenic driver.^37–39^ HCC development is mainly driven by inactivating mutations in various tumour-suppressor genes, which predispose hepatocytes to accumulate additional oncogenic changes that drive cancer initiation and tumorigenesis.^40,41^ The key genetic changes identified in HCC relevant to the properties of LCSCs are highlighted and discussed below.

### p53 mutation

Although p53 inactivation alone is insufficient to drive HCC tumorigenesis,^42^ its inactivation has been suggested to predispose hepatocytes to oncogenic transformation and acquisition of CSC properties. For example, expression of oncogenic c-myc in p53-deficient HCC cells significantly accelerates the growth of tumour xenografts and promotes self-renewal ability in terms of enhanced spheroid formation and increased expression of stemness genes.^43^ The inactivation of p53 through a mutation-independent mechanism has also been suggested to support LCSC properties.^44^ Liu et al.^44^ reported that cellular p53 can be degraded and suppressed by mitophagy, a specialised type of autophagy that specifically clears away mitochondria. Upon inhibition of mitophagy, the mitophagy-associated kinase phosphatase and tensin homologue (PTEN)-induced kinase 1 phosphorylates p53 and promotes its nuclear translocation; the nuclear p53 occupies the NANOG promoter and prevents its expression by OCT4 and SOX2, eventually blocking the LCSC properties and hepatocarcinogenesis.

### β-Catenin mutation

Canonical Wnt/β-catenin signalling has been observed in various LCSC models such as CD133^+^ and EpCAM^+^ LCSCs.^10,45^ As well as activating mutations in β-catenin, inactivating mutations in the protein subunits of the functional destruction complex for β-catenin, such as AXIN1 and glycogen synthase kinase 3 (GSK-3β), could result in Wnt activation. AXIN1 mutations are often recurrent in HCC and usually mutually exclusive to the activating β-catenin mutations.^39,41^

Serine/threonine kinase receptor-associated protein, a scaffold protein that has a role in Wnt pathway activation in colorectal cancer, has been found to be upregulated in HCC;^46^ its knockout downregulates the stem cell markers AXIN2 and LGR5 and upregulates other differentiation-associated genes. Similarly, a reduction in the cytoplasmic expression of RNA-binding motif on Y chromosome, which inhibits GSK-3β, significantly promotes β-catenin activation and contributes to the CSC phenotypes.^47^ These results suggest that Wnt pathway activity supports the hyperproliferation of HCC cells and more progenitor cell-like LCSC characteristics.

### Telomerase reverse transcriptase (TERT) promoter mutation

Mutations in the TERT gene promoter occur frequently in HCC (in >60% of cases). The expression and activity of TERT are tightly linked to stem cell functions by maintaining the self-renewal ability and ensuring the proliferation and differentiation efficiency. Mutation of its promoter could induce TERT expression, the de-differentiation of hepatocytes and the acquisition of LCSC properties during hepatocarcinogenesis. Indeed, in HCC cells expressing stemness-related proteins, such as cytokeratin-19, EpCAM and CD133, and having hTERT positivity, telomere length was significantly longer than in conventional HCC cells.^48^ Longer telomeres were associated with shorter overall and disease-free survival in patients with HCC.

### Viruses

Several studies have demonstrated the role of hepatitis viruses in contributing to liver cancer stemness properties. Integration of viral gene components into the genome of hepatocytes is a feature of chronic HBV infection. During the integration process, the hepatitis B X (HBx) gene is usually truncated at the C-terminus, resulting in the translation of a truncated HBx protein. Stable expression of truncated HBx protein enhanced the TIC population in immortalised normal liver cells, with a concomitant increase in STAT3 signalling and STAT3-driven CD133 expression.^49^ Furthermore, the truncated HBx-expressing cells show upregulated farnesoid X receptor (FXR) expression to promote CSC properties, which could be effectively reversed by a selective FXR inhibitor. Similarly, induced truncated HBx expression in HCC cell lines could promote CSC properties,^50^ such as the upregulation of NANOG.

The expression of EpCAM is normally suppressed in hepatocytes by the chromatin-modifying polycomb-repressive complex 2, but HBV replication within these cells induces EpCAM re-expression.^51^ EpCAM undergoes regulated intramembrane proteolysis during HBV replication, and the released intracellular domain, in complex with β-catenin, translocates to the nucleus to activate Wnt signalling, driving the expression of stemness marker genes and supporting chemoresistance.

Hepatitis C virus (HCV) is an RNA virus that exerts its biological activities without integrating into the host genome. In one study, the expression of a subgenomic replicon of HCV, the NS5B polymerase, is sufficient to promote the expression of a range of stemness genes.^52^

## Epigenetic factors relevant to stemness

A growing number of studies have been carried out to investigate the role of epigenetic modulation and non-coding epigenetic regulators, including microRNA and long non-coding RNA (lncRNA), in regulating liver cancer stemness properties; these studies are briefly discussed below.

### Epigenetic modulation

Dysregulated changes in epigenetic modulation—DNA methylation, microRNA and histone modifications—are linked with liver cancer stemness. For example, treating HCC cells with zebularine, a DNA methyltransferase inhibitor, led to global DNA hypomethylation and significantly increased the side population of liver cancer cells as defined by the functional characteristics of Hoechst-33342 dye exclusion. This population of cells displayed increased self-renewal properties and robust tumour-initiating abilities in a serial transplantation model,^53^ indicating the re-expression of genes involved in activating diverse oncogenic pathways.

Significant upregulation of lysine-specific demethylase-1 (LSD1) was reported in LGR5^+^ HCC cells.^31^ LSD1 reverses mono- and di-methylation of histone H3K4 in the promoter regions of negative regulators of Wnt pathway, including Prickle1 and APC, thereby inhibiting their expression, to promote β-catenin activation and support the CSC properties of LGR5^+^ cells. Furthermore, increased histone deacetylase (HDAC) activity in HCC, which in some cases might be associated with the expression of SALL4, has also been demonstrated to support liver cancer stemness.^6,54^

### MicroRNA and liver cancer stemness

MicroRNA profiling of LCSC and non-CSC cell populations has identified specific microRNAs that are associated with the expression of different CSC markers. For example, dysregulated levels of miR-130b, miR-142-3p and miR-1246p have been proposed to contribute to the stemness properties of CD133^+^ CSCs. Specifically, miR-130b and miR-1246p were overexpressed in HCC.^55,56^ miR-130b targets and downregulates the expression of tumour protein 53-induced nuclear protein 1, a pro-apoptotic tumour suppressor that triggers autophagy;^55^ while miR-1246p targets and downregulates the expression of AXIN2 and GSK-3β to promote Wnt signalling.^56^ By contrast, miR-142-3p directly targets and downregulates CD133 expression; miR-142-3p was found to be underexpressed in a CD133^+^ CSC population, and its re-expression antagonised CD133-mediated cancer stemness properties.^57^

Other miRNAs that are involved in CSC-marker-positive LCSCs have also been described. In EpCAM^+^ CSCs, abnormally low DNA methylation in the upstream region of the miR-200b/miR-200a/miR-420 microRNA cluster led to the enrichment of miR-429, which promotes the stemness properties of EpCAM^+^ TICs through the retinoblastoma-binding protein 4/E2F transcription factor 1/Oct4 axis.^58^ Interestingly, in cells expressing a high level of miR-429, this microRNA was also detectable in secreted microvesicles, suggesting a potential intercellular role in mediating intracellular gene expression.

### lncRNA and liver cancer stemness

**Fig. 1:**
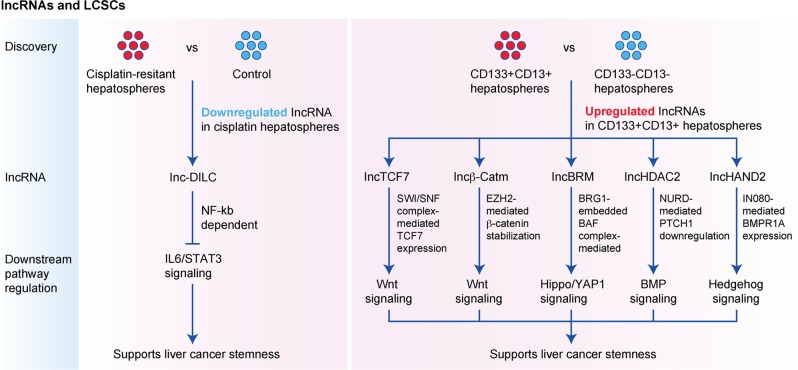
Identification of functionally important lncRNAs responsible for supporting liver cancer stemness. The use of microarray analyses for identifying lncRNAs downregulated in cisplatin-resistant hepatospheres or lncRNAs enriched in CD133^+^CD13^+^ hepatospheres. lncRNAs could support liver cancer stemness by modulating a range of signalling pathways critical in normal development and stem cells.

Over the past 5 years, a number of lncRNAs (non-coding RNAs with >200 nucleotides) showing dysregulated expression in HCC hepatospheres have been identified and reported to be involved in self-renewal and tumour progression in HCCs (Fig. 1). lnc-DILC—lncRNA downregulated in LCSCs—is commonly downregulated in cisplatin-resistant hepatospheres,^59^ and downregulation of lnc-DILC was found in HCC patients and significantly associated with shorter patient survival. Importantly, higher expression of stemness genes including interleukin (IL)-6, EpCAM and CD24 was observed in HCC with lnc-DILC downregulation, indicating a positive correlation between lnc-DILC downregulation and increased cancer stemness properties. Mechanistically, lnc-DILC directly interacts with the IL-6 promoter to block the expression of STAT3 stimulated by TNF-α and IL-1β and mediated by nuclear factor κB (NF-κB). Blocking the autocrine IL-6/STAT3 signal, in turn, suppresses LCSC expansion. By performing transcriptomic microarray profiling analysis comparing CD133^+^CD13^+^ hepatospheres with their CD133^−^CD13^−^ counterparts, the lncRNAs lncTCF7,^60^ lncBRM,^61^ lncβ-Catm,^62^ lncHDAC2^63^ and lncHAND2^64^ have been identified to be significantly upregulated in the presence of CD133 and CD13.

## Signalling pathways that function in cancer cell stemness

**Fig. 2:**
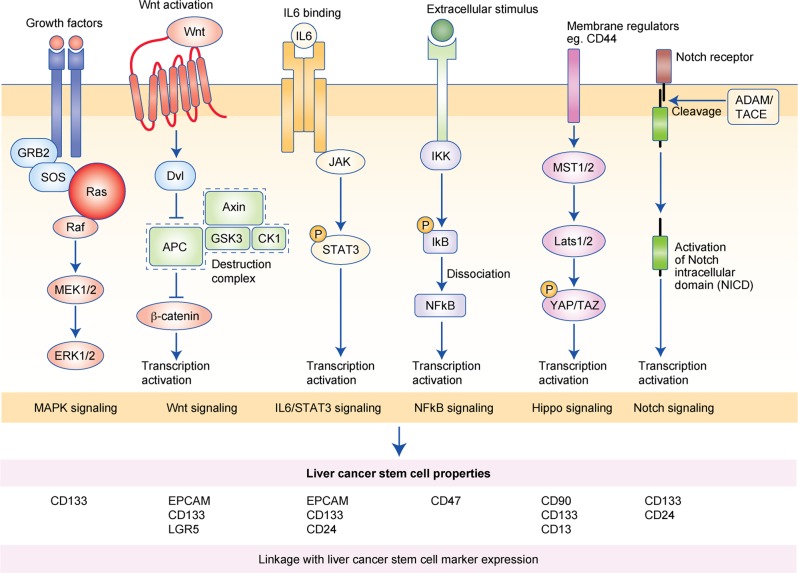
Key signalling pathways that support liver cancer stem cell properties and their association with liver cancer stem cell marker expression. Wnt/β-catenin and IL-6/STAT3 are the two main signalling pathways that support liver cancer stemness. Recent studies have provided additional evidence that MAPK signaling, NF-κB signaling, Hippo signaling, and Notch signaling are also playing critical roles in supporting liver cancer stemness and associated with the elevated expression of the indicated liver cancer stem cell markers.

Independent studies have shown that Wnt/β-catenin and IL-6/STAT3 are the two main signalling pathways that support liver cancer stemness, as mentioned above. However, other signalling pathways have also been reported to support liver cancer stemness, and representative examples are discussed below (Fig. 2).

### NF-κB signalling

CD47 was found to be upregulated in chemoresistant hepatospheres, and CD47 is preferentially expressed in liver TICs. The protease cathepsin S has been proposed to support the stemness properties of CD47^+^ HCC cells. Cathepsin S secreted by CD47^+^ hepatospheres binds to and activates the protease-activated receptor-2 (PAR2) to mediate autocrine signalling. Activation of PAR2 eventually forms a feed-forward signalling loop by promoting the nuclear translocation of NF-κB to further drive transcription and expression of cathepsin S.^28^

### Notch signalling

The role of Notch signalling in LCSCs has consistently been observed.^65,66^ iNOS has been demonstrated to promote Notch signalling specifically in CD24^+^CD133^+^ LCSCs by the activation of ADAM17/TACE, which mediates Notch cleavage in a soluble guanylate cyclase/cGMP/protein kinase G-dependent manner.^16^ The association of ADAM17/TACE upregulation with poorer overall survival suggests that Notch1 activation might play a role in supporting liver tumorigenesis by promoting LCSC properties. iNOS also increases the levels of rhomboid protein-2 (iRhom2), which functions as a positive regulator of Notch signalling by transporting ADAM17/TACE to the cell surface.

### Hippo signalling

Hippo signalling is important in the regulation of stem cell renewal and differentiation. Of its two downstream effectors, transcriptional coactivator with PDZ-binding motif (TAZ) and Yes-associated protein (YAP), TAZ was found to be predominantly expressed in HCC. Interestingly, the knockdown of TAZ attenuated growth under normal conditions but induced a compensatory upregulation of YAP together with an increase in the expression of the CSC marker CD90 in the presence of 5-fluorouracil, which contributes to chemoresistance.^67^ This result suggests that Hippo signalling might play an oncogenic role in HCC by conferring LCSC properties, and notably, that, when targeting Hippo signalling in HCC cells, simultaneous inhibition of TAZ and YAP should be considered to avoid potential compensatory actions.

### Mitogen-activated protein kinase (MAPK) signalling

In LCSCs, MAPK signalling was shown to be activated by intrinsic genetic alterations of tumour-suppressor proteins such as apoptosis-stimulating protein of p53 (ASPP2)^68^ or by extrinsic stimulation by growth factors such as hepatocyte growth factor (HGF) secreted by stromal cells.^69^ HGF secreted by cancer-associated fibroblasts (CAFs) transactivates the c-MET receptor and induces downstream MAPK signalling to activate FRA1-mediated transcription of hairy/enhancer-of-split related with YRPW motif 1 (HEY1) to support the cancer initiation properties in LCSCs. In 2018, Chan et al.^70^ reported the specific downregulation of protein arginine methyltransferase 6 (PRMT6), a chromatin modifier, in CD133^+^ LCSCs. PRMT6 can also perform a chromatin-modifying independent function by methylating and binding to the R100 residue of c-Raf kinase in the cytoplasm, which inhibits the interaction of Ras with c-Raf to abolish MAPK pathway activation; the downregulation of PRMT6 therefore alleviates this inhibition. This suggests a novel mechanism—through posttranslational modification of key signal transducers in the pathway—by which LCSCs maintain MAPK signalling.

## Plasticity of cancer stemness

**Fig. 3:**
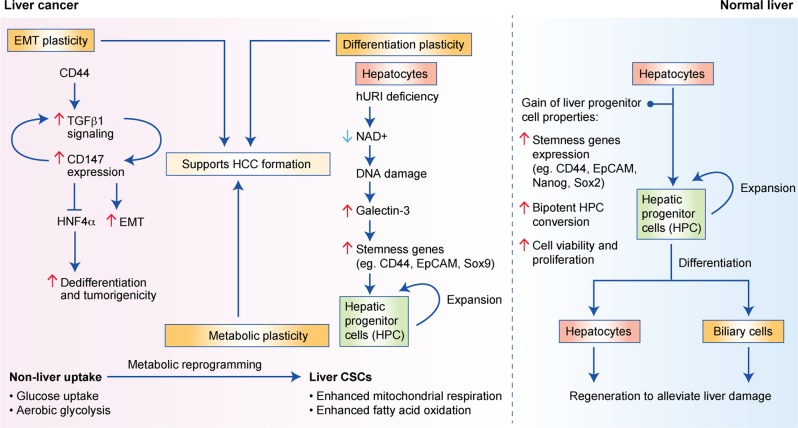
The plasticity of LCSCs refers to the acquisition of the necessary changes in cell states to facilitate the adaptation of LSCSs to the tumour microenvironment. This includes plasticity in the form of EMT, metabolic reprogramming and differentiation plasticity. EMT refers to the change from epithelial to mesenchymal states of the cells. Metabolic reprogramming shifts the reliance on glucose and aerobic glycolysis to enhanced mitochondrial respiration and fatty acid oxidation. Differentiation plasticity allows the expansion of hepatic progenitor cells (HPCs) and promotes stemness gene expression. All of these support HCC formation.

Plasticity is regarded as the ability of cells to reversibly acquire cell fates or phenotypes of other cell types in a tissue-restrictive manner and is distinct from the reprogramming of differentiated cells back to the primitive pluripotent stem cell state.^1^ Any interconversion between different differentiated cell states or phenotypes (trans-differentiation)^1,71^ or de-differentiation of differentiated cells to progenitor cells^1^ should be considered as plasticity. Below, we focus on some examples of plasticity in relation to tumour initiation, such as epithelial–mesenchymal transition (EMT), differentiation plasticity and metabolic plasticity in CSCs (Fig. 3).

### Epithelial-to-mesenchymal transition

EMT is the transition from an epithelial state to a mesenchymal state of cells. It can be induced by TGF-β in HCC,^72^ and one study found that TGF-β1 and CD147 formed a self-sustaining signalling, in which CD147 increased TGF-β1 expression, whereas TGF-β1 promoted the expression of CD147, leading to a positive correlation between TGF-β1 and CD147 in the DEN-induced HCC mouse model.^72^ The overexpression of CD147 also promoted the loss of E-cadherin to enhance epithelial plasticity towards a more mesenchymal state.^72^ Such self-sustained TGF-β1 signalling inhibits hepatocyte nuclear factor 4α (HNF4α), a marker for differentiated MHs, to promote de-differentiation of hepatocytes. The overexpression of HNF4α and the TGF-β1-inhibitory Smad7 suppressed tumorigenicity of HCC cells in a CD147-knockdown background.^72^ This study showed a role of TGF-β1 and CD147 in a positive relationship to promote cancer stemness and the plasticity in EMT in HCC. Furthermore, CD44 was required to promote TGF-β-induced EMT-related gene expression since sorted CD44-postive but not CD44-negative PLC/PRF/5 cells exhibit EMT-related gene induction in the presence of TGF-β.^73^ The CD44-positive cells also showed enhanced migratory and invasive ability.^73^ This indicated that CD44, which is one marker for cancer stemness, was involved in the plasticity of cancer cells to transit into the more aggressive mesenchymal state.

However, whether EMT is firmly related to cancer stemness is controversial. In HPCs, TGF-β can not only induce EMT but also inhibit proliferation. However, HPCs that have adapted to chronic TGF-β exposure were resistant to apoptosis induced by serum starvation and acute TGF-β exposure^74^ and were able to alleviate liver damage induced by carbon tetrachloride.^74^ By making use of c-Met-knockout HPCs, it was found that c-Met signalling was required for the acquisition of enhanced viability and regenerating power resulted from TGF-β over-exposure but was not needed for the EMT phenotypes induced by TGF-β.^74^ The growth arrest resulted from c-Met knockout was accompanied by increased expression of cyclin-dependent kinase inhibitors p15 and p19 and increased senescence and reactive oxygen species (ROS).^74^ Such c-Met-mediated proliferation in chronically TGF-β-exposed HPCs was a post-EMT event as the c-Met-promoted phenotype also required the participation of an EMT marker, Twist1.^74^ Overall, there exists a complicated interplay between TGF-β and HGF/c-Met signalling in HPCs: TGF-β promotes plasticity by means of EMT, whereas HGF/c-Met signalling helps the expansion of HPCs by suppressing the growth-inhibitory effects of TGF-β. This suggests separate roles of plasticity of EMT and stemness in normal HPCs as chronic TGF-β exposure did not enhance the expression of stemness proteins.^74^

On the other hand, a cocktail of HGF, A83-01 (a TGF-β inhibitor) and CHIR99021 (a GSK-3β inhibitor) converted hepatocytes from healthy human liver donor cells into bipotential HPCs,^75^ which showed enhanced expression of markers of pluripotency and were capable of differentiation into both hepatocytes and biliary epithelial cells. This implies inhibiting TGF-β signalling would increase the plasticity of normal hepatocytes towards bipotential HPCs, whereas it is intriguing that activating TGF-β signalling can induce plasticity towards a more mesenchymal state in HCC cell models. This result might implicate a context-dependent role of TGF-β in the plasticity and stemness of cancerous liver cells as compared with normal liver cells.^76^ It is also in keeping with the complex role of TGF-β as a tumour suppressor and a pro-metastatic factor, depending on the context and stages of HCC development.^77,78^ More, improved liver cancer models are needed to elucidate this further.

### Differentiation plasticity

The human unconventional prefoldin RPB5 interactor (hURI)-tetOFF^hep^ mouse HCC model is a model in which hepatocyte-specific NAD^+^-deficit-induced DNA damage mimics human hepatocarcinogenesis through multistep liver tumour development.^79^ As Sox9 is the marker for HPCs, crossing hURI-tetOFF^hep^ mice with Sox9^IRES-EGFP^ mice can result in tumours with the HPC-derived cells labelled as enhanced green fluorescent protein (EGFP)-positive cells, which were found to be positive of EpCAM, CD133 and CD44 protein expression, thus indicating cancer stemness in the tumours derived from the bipotent HPCs in hepatocarcinogenesis. Similar finding was obtained by crossing hURI-tetOFF^hep^ mice with Sox9^IRES-CreERT2^ and the serum albumin (SA)^CreERT2^ mice, respectively, which both contained the R26-stop-EYFP reporter to label the HPCs and hepatocytes, respectively, with enhanced yellow fluorescent protein (EYFP) upon the Cre-recombination. Lineage tracing experiments for both crossed mouse models showed that HPCs and hepatocytes could derive HCC-containing EYFP-positive and EYFP-negative HCC cells within the respective tumours. Galectin-3 was identified to be highly expressed in the hURI-expressing cells, and conditioned medium from galectin-3-knockdown hepatocytes failed to promote the expression of stemness genes in, and the expansion of, HPCs. Heterozygous knockout of galectin-3 also showed a reduction in the number of HPCs and hepatic tumour formation. This indicates that HCC can be derived from both HPCs and hepatocytes and plasticity across these cell types might be related to cancer stemness and tumour initiation, depending on the context of HCC initiation. Further study will be needed to delineate the plasticity of HPCs and hepatocytes in relation to cancer stemness in improved liver cancer models.

### Metabolic plasticity

Plasticity in the metabolic reprogramming of HCC cells has been investigated using the Dt81Hepa1-6 cell line. This cell line is derived from the in vivo passage of the Hepa1-6 cell line in C57BL/6 mice^80^ and is more tumorigenic than its parental counterpart, potentially owing to its ability to undergo metabolic adaptation. In the presence of high glucose, Dt81Hepa1-6 cells have enhanced glucose uptake and aerobic glycolysis as well as fatty acid synthesis, compared with the parental cell line.^80^ This metabolic change might enable the Dt81Hepa1-6 cells to adapt to glucose-restricted conditions better than the parental cell line by using fatty acids as an alternative energy source.^80^

In another cell-sorting study, Wei et al.^81^ found an increased expression of proteins conferring mitochondrial biogenesis and fusion, but a reduced expression of those involved in mitochondria fission in LCSCs (Nanog^high^ cells) compared with their Nanog^low^ non-CSC counterparts. As mitochondria are a population of dynamic organelles with opposing fusion and fission processes to regulate mitochondrial function, mitochondrial fusion can promote oxidative phosphorylation while fission inhibits it. Complex I of the mitochondrial respiratory chain is required for the generation of NAD^+^ for mitochondrial function. One component of complex I is mitochondrial ribosomal protein S5 (MRPS5); its expression, at high levels, was associated with poorer HCC patient survival,^81^ whereas its knockdown increased mitochondrial membrane potential, decreased oxygen consumption, suppressed tumorigenicity and decreased tumour growth.^81^ Further investigation showed that sirtuin 1 (SIRT1), a deacetylase that is expressed at high levels in LCSCs, reduced the acetylation of MRPS5, thus reducing its nuclear localisation and the expression of glycolysis-related genes.^81^ This, in turn, promoted the localisation of MRPS5 within the cytoplasm and enhanced mitochondrial function and aerobic respiration. Hence, these results indicate that SIRT1/MRPS5 is critical in controlling the metabolic switch of the cells to mitochondrial aerobic respiration from relying more on glycolysis during the gain of cancer stemness phenotypes.

These studies indicate the presence of a switch to mitochondrial aerobic respiration for HCC cells to undergo de-differentiation into HCC cells with a cancer stemness phenotype, highlighting the flexibility to use an alternative energy source when encountering glucose restriction. Further investigation of such metabolic reprogramming in sorted HCC cells according to cancer stemness markers will be needed to further elucidate the mechanism(s).

## Cancer stemness and the tumour microenvironment

**Fig. 4:**
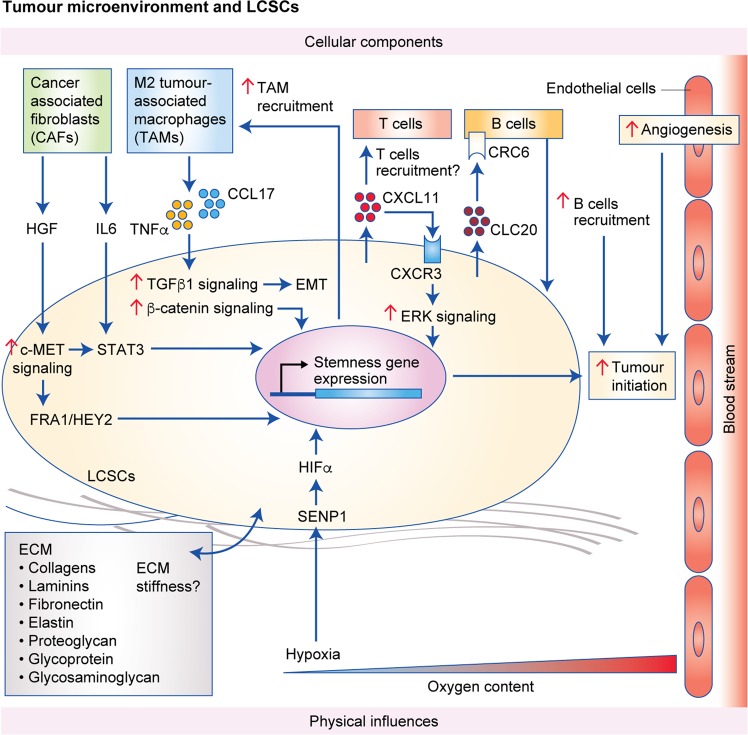
Key cellular components (upper part) and physical influences (lower part) in the liver tumour microenvironment that support stemness gene expression and tumour initiation. Cellular components include CAFs, TAMs, T cells, B cells and the respective secreted cytokines and factors. The physical components consist of ECM and the hypoxic environment. All of these play roles in promoting stemness gene expression to drive the tumour-initiation activity of LCSCs.

The tumour microenvironment in HCC comprises the extracellular matrix (ECM), which contains a large variety of cells other than liver cancer cells, and is believed to contribute to the niche for cancer stemness. In this section, we divide the tumour microenvironment into physical and cellular components to facilitate discussion (Fig. 4).

### Physical influences

The ECM is a complex non-cellular three-dimensional network of collagens, laminins, fibronectin, elastin, proteoglycans, glycosaminoglycans and other glycoproteins, which provides structural and biochemical support.^82^ According to different studies, both low^83^ and high^84^ ECM stiffness have been implicated to promote cancer stemness. There are no mechanistic studies for how low ECM stiffness promotes cancer stemness, while high ECM stiffness activates the Akt/mammalian target of rapamycin (mTOR) signalling pathway in an integrin β1-dependent manner to promote cancer stemness. Whether variation in these findings is due to the different HCC cell lines used and differing ranges of matrix stiffness applied requires further investigation. In addition, the γ2 chain of laminin-332^85^ and type 1 collagen α chains (COL1A1)^82^ have both been suggested to promote cancer stemness, and further functional studies with tumorigenicity assays are needed to validate these results.

Hypoxia has been proposed to arise in solid tumours and occurs particularly frequently in HCC, due to its rapid growth.^86^ Hypoxic conditions were shown to enhance the expression of CD44 and Oct4 and EMT-related genes, as well as promoting vascular mimicry in HepG2 cells, compared with normoxia.^87^ Our previous study also found that hypoxia increased the transcription of sentrin-specific protease 1 (SENP1), a member of the de-SUMOylation protease family, by inducing hypoxia-inducible factor (HIF)-1α, causing SENP1 to repress the SUMOylation of HIF-1α and thereby increase its stabilisation and transcriptional activity; HIF-1α is strongly implicated in the maintenance of stemness,^88,89^ and our study revealed that HIF-1α and SENP1 co-operated in a positive manner.^86^

### Cellular components

Cellular components in the microenvironment include CAFs, tumour-associated macrophages (TAMs) and many other immune cells. Previous studies have demonstrated that CAFs increase the tumorigenicity of HCC cells. Conditioned medium from CAFs promotes sphere formation,^69,90,91^ the expression of stemness-related genes, such as CD24,^90^ CD44,^69^ CD47,^69^ CD90,^69^ Nanog,^90,91^ Oct4^90,91^ and Sox2,^90,91^ and chemoresistance to cisplatin and doxorubicin.^69^ It was further demonstrated that CAF-induced cancer stemness in HCC cells was stimulated by HGF^69,90^ via the c-MET/FRA1/HEY1 signalling axis (see above) or the c-MET/STAT3 axis or by IL-6 via STAT3 in CD24-positive HCC cells.^90^ It is also interesting that conditioned medium derived from peritumoural CAFs enhanced the metastatic properties and tumorigenicity of HCC cells to a greater extent than did conditioned medium from CAFs from within the tumour. The increase in metastatic properties and tumorigenicity was found to be induced by several cytokines in the conditioned medium, including IL-6, C-C motif chemokine ligand 2 (CCL2) and chemokine (C-X-C motif) ligand 1 (CXCL1). HCC cells upregulated their expression of C-C chemokine receptor type 2 (CCR2) and C-X-C motif chemokine receptor 1 (CXCR1), and also had EpCAM expression,^92^ indicating that peritumoural fibroblasts could potentially recruit and maintain CSCs.

Conditioned medium from polarised M2 TAMs promoted migration,^93,94^ tumour growth,^93,94^ sphere formation,^93^ expression of stemness proteins,^93,94^ a side population of cancer stemness,^93^ TGF-β/p-Smad^93^ and β-catenin^93,94^ signalling pathways and EMT^93,94^ in HCC cell lines.^93^ CCL-17 and TNF-α, which are overexpressed in HCC^93^ and implicated in HCC development,^95,96^ respectively, were found be the functional components secreted by TAMs in promoting these phenotypes. Another study used fluorescence-activated cell sorting to identify a small subpopulation of cells in HCC that had low proteasomal activity, a feature of stem cells, and further demonstrated that these cells had increased CD44 marker expression and tumorigenicity and could facilitate macrophage migration in vitro and metastatic potential in vivo through macrophage recruitment.^97^ This indicates that HCC cells with cancer stemness properties might cooperate with macrophages to facilitate tumour initiation.

CXCL11, a cytokine involved in recruiting activated T cells to inflammation sites, was found to be overexpressed in HCC and in α2δ1^+^ HCC TICs.^98^ Knockdown of CXCL11 suppressed the expression of stemness genes, sphere formation, tumorigenicity and chemoresistance to doxorubicin in the α2δ1^+^ HCC TICs, whereas overexpression reversed these phenotypes.^98^ CXCR3, the receptor for CXCL11, stimulates downstream extracellular signal-regulated kinase 1/2 signalling to promote cancer stemness in HCC cells,^98^ indicating a positive role for inflammatory CXCL11 in HCC cancer stemness and progression. Another study of chemokines in HCC found that CCL20 was overexpressed in HCC and that a high serum CCL20 level was associated with poorer disease-free and overall survival.^99^ The receptor for CCL20, CCR6, was expressed in intrahepatic CD45-positive tumour-infiltrating immune cells, and metastatic HCC tissues had a higher expression of CCR6 than non-metastatic tissues.^99^ A blocking antibody against CCL20 reduced the recruitment of CD19^+^CD5^+^ B cells, which expressed high levels of CXCR6, towards HCC cells in chemotaxis assays.^99^ An anti-CCL20 antibody also reduced tumour incidence, growth and metastasis of HCC cells in subcutaneous HCC mouse xenograft models with an immunocompetent background. A tumour-promoting role for CCL20-induced B cell recruitment was subsequently confirmed.^99^ These results suggest that chemokines released by HCC cells can help to recruit tumour-promoting immune cells to promote angiogenesis and tumour initiation in HCC but whether angiogenesis and tumour initiation essentially occur in sequential order and whether the recruited B cells enhance stemness genes/markers in HCC cells in the above mouse models needs further investigation.

## Cancer stemness and chemoresistance

CSCs acquire chemoresistance through multiple mechanisms. First, CSCs can increase drug efflux by upregulating the expression of transmembrane exporters such as ATP-binding cassette superfamily G member 2 (ABCG2) (driven by Oct4),^100^ ABCB5 (driven by granulin-epithelin precursor)^101^ and multi-drug resistance transporter-1 (driven by c-Myc, Nanog or IL-8)^102–104^ to prevent drug exposure. The nature of the transporter used by the CSCs depends on the genetic background and the underlying oncogenic drivers in the CSCs.^102^ Second, LCSCs can activate pro-survival signalling pathways such as those mediated by Akt and BCL-2 while suppressing apoptotic pathways to antagonise the cytotoxic effects of anticancer drugs.^11,105^ Blocking the pro-survival signalling pathways can re-sensitise the cells to the drug treatment. Third, tumour microenvironment-related factors such as hypoxia or intercellular interactions (e.g. with hepatic stellate cells and CAFs) through paracrine signalling can confer CSC properties and chemoresistance upon liver cancer cells.^69,86,106^

Expression profiling of sorafenib-resistant hepatospheres revealed CD47 as a significantly upregulated tumour-initiation marker compared with control parental HCC cells.^107^ CD47 knockdown or inhibition by neutralising antibodies re-sensitised the sorafenib-resistant cells to sorafenib treatment. The expression of CD24 in HCC cells has also been shown to be positively correlated with sorafenib resistance^108^ through the activation of protein phosphatase 2A, which induces inactivation of the mTOR/Akt pathway. Inactivation of mTOR enhances the level of autophagy to mediate sorafenib resistance. Another protein that induces autophagy is annexin A3 (ANXA3), the levels of which are elevated in sorafenib-resistant HCC cells and patient-derived xenograft samples. These cells and samples are also enriched in CD133, which was previously identified to mark a subset of CSCs in HCC, implicating ANXA3 in the control of stemness and resistance towards sorafenib and regorafenib.^109,110^ ANXA3 activates autophagy for cell survival and specifically suppresses protein kinase Cδ/p38-mediated apoptosis induced by stress. Accordingly, combining an anti-ANXA3 monoclonal antibody with sorafenib or regorafenib has been demonstrated to effectively overcome resistance to these compounds.^104^

## Specific therapeutic targeting of liver cancer stemness

Targeted therapy (using antibodies or inhibitors) of LCSC markers can effectively eliminate LCSCs, and this approach has previously been reported in preclinical animal models (reviewed in ref. ^111^) However, directly targeting specific LCSC markers to eradicate CSCs is challenging, as some of the markers are also expressed in other organs, in which they serve important physiological functions. Instead, there are other potential directions that can be explored to specifically target liver cancer stemness, as outlined below.

### Reversion of the dysregulated biological processes underlying liver cancer stemness

Small interfering RNA-based synthetic lethality screening in EpCAM^+^ LCSCs uncovered mitochondrial-processing peptidase subunit β, which, upon inhibition, not only significantly suppressed EpCAM expression and Wnt/β-catenin signalling but also induced apoptosis in EpCAM^+^ LCSCs via intracellular ROS accumulation.^112^ Similarly, inhibition of glutaminase-1, an enzyme specifically expressed in the mitochondrial matrix to support LCSC properties, could lead to ROS accumulation and suppression of the Wnt/β-catenin pathway.^113^ These findings suggest that disrupting the redox balance might be a useful approach to target LCSCs. Furthermore, chromodomain helicase DNA-binding-protein 4 (CHD4), which possesses HDAC and poly-ADP ribose polymerase (PARP) activities, was also found to be enriched in EpCAM^+^ LCSCs, where it epigenetically regulates gene expression and the DNA damage response, contributing to stemness properties and chemoresistance in response to epirubicin treatment.^114^ This finding could open up potential therapeutic opportunities using deacetylase and PARP inhibitors, the latter of which has already been used in breast cancer treatment.

Another potential strategy is to target the metabolic vulnerabilities of LCSCs. Three independent studies have identified stearoyl-CoA desaturase (SCD1), an enzyme responsible for the synthesis of mono-unsaturated fatty acids (MUFAs), as a critical player in mediating HCC tumorigenesis and CSC properties.^115–117^ SCD1 is significantly upregulated in hepatic stellate cells and HCC cells from patients, and its expression is associated with more advanced HCC stage and shorter disease-free survival. SCD1 promotes liver cancer stemness through the synthesis of MUFAs, which can stabilise β-catenin, thereby inducing Wnt/β-catenin signalling, as well as generating a feed-forward loop to elevate SCD1 expression.^115^ SCD1 can also contribute to sorafenib resistance by inhibiting the endoplasmic reticulum stress-induced unfolded protein response.^116^ Importantly, suppression of SCD1 by a specific inhibitor not only sensitises the cells to sorafenib treatment but is also sufficient to suppress liver fibrosis and liver tumorigenesis.

### Inducing differentiation of LCSCs

IL-8 has been found to confer LCSC properties and is associated with a poorer prognosis in HCC patients. Its signalling network has also been found to be significantly upregulated in CD133^+^ LCSCs, in which enhanced IL-8 secretion conferred a greater ability to support self-renewal and angiogenesis.^118^ As the mouse homologue of IL-8, mCXCL1, has been demonstrated to be required for maintaining the quiescent state of LCSCs in an mTOR complex 1 (mTORC1)-dependent manner,^103^ blocking IL-8 or mTORC1 could promote the differentiation of LCSCs, which might serve as a novel strategy to eliminate stem cell-like CSC populations. Similarly, high-dose treatment with bone morphogenetic protein 4, which has an important role in hepatogenesis and hepatic stem cell differentiation, could promote the differentiation of CD133^+^ LCSCs and thus suppress CSC-related functions.^119^

## The search for, and development of, novel compounds to directly target LCSCs

### Natural compounds

Natural compounds isolated from plants might possess anti-LCSC properties. For example, Lup-20(29)-en-3b-ol (Lupeol), a triterpene derived from fruits and vegetables, can suppress CD133^+^ LCSC populations and sensitise HCC cells to chemotherapeutic agents by modulating PTEN.^120^ In addition, baicalein, a type of flavonoid isolated from plant roots, can inhibit SAR1B GTPase, which is required for autophagy, to specifically sensitise LCSCs, but not normal cells, to mTORC1 inhibition.^121^

### Synthetic compounds

The presence of LCSCs is associated with HCC recurrence. However, a vitamin A-like synthetic compound, acyclic retinoid (ACR), has been demonstrated to significantly reduce the extent of HCC recurrence.^122^ ACR suppresses the expression of MYCN, a basic-loop-helix transcription factor important for supporting LCSC properties and underlying Wnt/β-catenin signalling. EpCAM^+^ LCSCs were demonstrated to have increased levels of MYCN expression and showed sensitivity to ACR, indicating that ACR treatment might serve as an effective strategy to target and remove EpCAM^+^ LCSCs.

## LCSC-targeted immunotherapy

There is a paradigm shift in cancer therapy from targeted therapy to harnessing the immune system to eradicate the cancer cells. Nivolumab and Pembrolizumab are the two anti-programmed cell death protein 1 (PD1) antibodies approved as the second-line agents for patients with advanced HCC refractory to sorafenib. Although the overall response rate of anti-PD1 treatment in HCC patients remains to be improved, the complete remission of the liver tumours in some of the anti-PD1-treated patients suggested that immunotherapy could also be a useful strategy to target LCSCs. Indeed, LCSCs have been shown to utilise PD1/PD ligand 1 (PD-L1)-dependent and PD-L1-independent mechanisms to evade immunosurveillance. For example, SOX2, a cancer stemness-related transcription factor being elevated in HCC tissues, has been shown to directly bind to the consensus sequence of the PD-L1 promoter and drives PD-L1 transcription.^123^ On the other hand, a positive correlation between the expression of IL-6 and PD-L1 has also been observed in HCC patients.^124^ Activation of the IL-6/Janus kinase-1 pathway could support the protein glycosylation and stabilisation of the PD-L1 receptor and facilitates its immunosuppressive function. Besides the PD1/PD-L1-dependent mechanism, recurrent HCC cells that expressed a high level of stemness marker genes tend to express a lower level of HLA molecules and resulting potentially in less neoantigen presentation, which facilitate their evasion from adaptive immunity.^125^ To overcome this, innate immune cells such as cytokine-induced killer cells, a form of CD3^+^ and CD56^+^ natural killer (NK) cells, which targets and eradicates LCSCs through an NK group 2D ligand-dependent mechanism might be employed instead.^126^

In addition to immune checkpoint blockade, a number of studies have developed and assessed the therapeutic potential of genetically engineered immune cells targeting liver CD133^+^ CSCs. As mentioned above, ANXA3 was found to be preferentially expressed in LCSCs and especially enriched in the CD133^+^ cell population. Dendritic cells transfected with ANXA3 are capable of inducing more functionally active T cells able to recognise and eradicate CD133^+^ LCSCs.^89^ Last year, a Phase I clinical trial was reported to assess the toxicity and treatment efficacy of CD133-targeted chimeric antigen receptor (CAR-T) cells in a patient cohort consisting of HCC, pancreatic and colorectal patients.^127^ It was demonstrated that CD133-targeted CAR-T cells showed good specificity in eliminating CD133^+^ tumours. More than half of the patients achieved a stable disease and a small number of patients even achieved complete remission after the CD133-targeted CAR-T cell infusion. Although the CD133-targeted CAR-T cell infusion was tolerated in cancer patients, its toxicity leading to a decrease in haemoglobin, lymphocytes and platelets remains a challenge to overcome before repeated and extended infusion is possible. Nevertheless, CD133-targeted therapy against LCSCs by genetically engineered immune cells could potentially serve as an alternative treatment strategy for late-stage HCC patients who are refractory to the currently available treatment options.

## Future perspectives

The state and survival of CSCs are controlled by various intrinsic factors, such as genetic and epigenetic alterations, as well as extrinsic physical and cellular elements that are derived from the tumour microenvironment. Targeting CSCs and their niches is a feasible approach to treating cancers, and several clinical trials targeting CSCs in other cancers, such as using dendritic cell immunotherapy against CSCs (NCT03548571), are ongoing. The ultimate goal of investigating the properties of LCSCs and LCSC cross-talk within its niches is to translate our knowledge into effective therapeutics against HCC.

## Data Availability

Not applicable.

## References

[1] Huch M, Dolle L (2016). The plastic cellular states of liver cells: are EpCAM and Lgr5 fit for purpose?. Hepatology.

[2] Tirino V, Desiderio V, Paino F, De Rosa A, Papaccio F, La Noce M (2013). Cancer stem cells in solid tumors: an overview and new approaches for their isolation and characterization. FASEB J..

[3] O’Connor ML, Xiang D, Shigdar S, Macdonald J, Li Y, Wang T (2014). Cancer stem cells: a contentious hypothesis now moving forward. Cancer Lett..

[4] Kreso A, Dick JE (2014). Evolution of the cancer stem cell model. Cell Stem Cell.

[5] Rizzino A (2013). Concise review: The Sox2-Oct4 connection: critical players in a much larger interdependent network integrated at multiple levels. Stem Cells.

[6] Zeng SS, Yamashita T, Kondo M, Nio K, Hayashi T, Hara Y (2014). The transcription factor SALL4 regulates stemness of EpCAM-positive hepatocellular carcinoma. J. Hepatol..

[7] Lee TK, Castilho A, Cheung VC, Tang KH, Ma S, Ng IO (2011). CD24(+) liver tumor-initiating cells drive self-renewal and tumor initiation through STAT3-mediated NANOG regulation. Cell Stem Cell.

[8] Visvader JE, Lindeman GJ (2008). Cancer stem cells in solid tumours: accumulating evidence and unresolved questions. Nat. Rev. Cancer.

[9] Medema JP (2013). Cancer stem cells: the challenges ahead. Nat. Cell Biol..

[10] Ma S, Chan KW, Hu L, Lee TK, Wo JY, Ng IO (2007). Identification and characterization of tumorigenic liver cancer stem/progenitor cells. Gastroenterology.

[11] Ma S, Lee TK, Zheng BJ, Chan KW, Guan XY (2008). CD133+ HCC cancer stem cells confer chemoresistance by preferential expression of the Akt/PKB survival pathway. Oncogene.

[12] Yamashita T, Ji J, Budhu A, Forgues M, Yang W, Wang HY (2009). EpCAM-positive hepatocellular carcinoma cells are tumor-initiating cells with stem/progenitor cell features. Gastroenterology.

[13] Sun YF, Xu Y, Yang XR, Guo W, Zhang X, Qiu SJ (2013). Circulating stem cell-like epithelial cell adhesion molecule-positive tumor cells indicate poor prognosis of hepatocellular carcinoma after curative resection. Hepatology.

[14] Haraguchi N, Ishii H, Mimori K, Tanaka F, Ohkuma M, Kim HM (2010). CD13 is a therapeutic target in human liver cancer stem cells. J. Clin. Invest..

[15] Yamada T, Abei M, Danjoh I, Shirota R, Yamashita T, Hyodo I (2015). Identification of a unique hepatocellular carcinoma line, Li-7, with CD13(+) cancer stem cells hierarchy and population change upon its differentiation during culture and effects of sorafenib. BMC Cancer.

[16] Wang R, Li Y, Tsung A, Huang H, Du Q, Yang M (2018). iNOS promotes CD24(+)CD133(+) liver cancer stem cell phenotype through a TACE/ADAM17-dependent Notch signaling pathway. Proc. Natl Acad. Sci. USA.

[17] Fernando J, Malfettone A, Cepeda EB, Vilarrasa-Blasi R, Bertran E, Raimondi G (2015). A mesenchymal-like phenotype and expression of CD44 predict lack of apoptotic response to sorafenib in liver tumor cells. Int. J. Cancer.

[18] Dang H, Steinway SN, Ding W, Rountree CB (2015). Induction of tumor initiation is dependent on CD44s in c-Met(+) hepatocellular carcinoma. BMC Cancer.

[19] Xia H, Kong SN, Chen J, Shi M, Sekar K, Seshachalam VP (2016). MELK is an oncogenic kinase essential for early hepatocellular carcinoma recurrence. Cancer Lett..

[20] Zhou S, Du R, Wang Z, Shen W, Gao R, Jiang S (2019). TLR4 increases the stemness and is highly expressed in relapsed human hepatocellular carcinoma. Cancer Med..

[21] Chen CL, Tsukamoto H, Liu JC, Kashiwabara C, Feldman D, Sher L (2013). Reciprocal regulation by TLR4 and TGF-beta in tumor-initiating stem-like cells. J. Clin. Invest..

[22] Terris B, Cavard C, Perret C (2010). EpCAM, a new marker for cancer stem cells in hepatocellular carcinoma. J. Hepatol..

[23] Yamashita T, Forgues M, Wang W, Kim JW, Ye Q, Jia H (2008). EpCAM and alpha-fetoprotein expression defines novel prognostic subtypes of hepatocellular carcinoma. Cancer Res..

[24] Yamashita T, Budhu A, Forgues M, Wang XW (2007). Activation of hepatic stem cell marker EpCAM by Wnt-beta-catenin signaling in hepatocellular carcinoma. Cancer Res..

[25] Yamashita T, Honda M, Nakamoto Y, Baba M, Nio K, Hara Y (2013). Discrete nature of EpCAM+ and CD90+ cancer stem cells in human hepatocellular carcinoma. Hepatology.

[26] Liu S, Li N, Yu X, Xiao X, Cheng K, Hu J (2013). Expression of intercellular adhesion molecule 1 by hepatocellular carcinoma stem cells and circulating tumor cells. Gastroenterology.

[27] Cheng BY, Lau EY, Leung HW, Leung CO, Ho NP, Gurung S (2018). IRAK1 augments cancer stemness and drug resistance via the AP-1/AKR1B10 signaling cascade in hepatocellular carcinoma. Cancer Res..

[28] Lee TK, Cheung VC, Lu P, Lau EY, Ma S, Tang KH (2014). Blockade of CD47-mediated cathepsin S/protease-activated receptor 2 signaling provides a therapeutic target for hepatocellular carcinoma. Hepatology.

[29] Huch M, Dorrell C, Boj SF, van Es JH, Li VS, van de Wetering M (2013). In vitro expansion of single Lgr5+ liver stem cells induced by Wnt-driven regeneration. Nature.

[30] Fukuma M, Tanese K, Effendi K, Yamazaki K, Masugi Y, Suda M (2013). Leucine-rich repeat-containing G protein-coupled receptor 5 regulates epithelial cell phenotype and survival of hepatocellular carcinoma cells. Exp. Cell Res..

[31] Lei ZJ, Wang J, Xiao HL, Guo Y, Wang T, Li Q (2015). Lysine-specific demethylase 1 promotes the stemness and chemoresistance of Lgr5(+) liver cancer initiating cells by suppressing negative regulators of beta-catenin signaling. Oncogene.

[32] Tsui YM, Sze KM, Tung EK, Ho DW, Lee TK, Ng IO (2017). Dishevelled-3 phosphorylation is governed by HIPK2/PP1Calpha/ITCH axis and the non-phosphorylated form promotes cancer stemness via LGR5 in hepatocellular carcinoma. Oncotarget.

[33] Yamamoto Y, Sakamoto M, Fujii G, Tsuiji H, Kenetaka K, Asaka M (2003). Overexpression of orphan G-protein-coupled receptor, Gpr49, in human hepatocellular carcinomas with beta-catenin mutations. Hepatology.

[34] Benabou E, Salame Z, Wendum D, Lequoy M, Tahraoui S, Merabtene F (2019). Insulin receptor isoform A favors tumor progression in human hepatocellular carcinoma by increasing stem/progenitor cell features. Cancer Lett..

[35] Lo RC, Leung CO, Chan KK, Ho DW, Wong CM, Lee TK (2018). Cripto-1 contributes to stemness in hepatocellular carcinoma by stabilizing Dishevelled-3 and activating Wnt/beta-catenin pathway. Cell Death Differ..

[36] Liu L, Dai Y, Chen J, Zeng T, Li Y, Chen L (2014). Maelstrom promotes hepatocellular carcinoma metastasis by inducing epithelial-mesenchymal transition by way of Akt/GSK-3beta/Snail signaling. Hepatology.

[37] Sung WK, Zheng H, Li S, Chen R, Liu X, Li Y (2012). Genome-wide survey of recurrent HBV integration in hepatocellular carcinoma. Nat. Genet..

[38] Kan Z, Zheng H, Liu X, Li S, Barber TD, Gong Z (2013). Whole-genome sequencing identifies recurrent mutations in hepatocellular carcinoma. Genome Res..

[39] Cancer Genome Atlas Research Network. (2017). Comprehensive and integrative genomic characterization of hepatocellular carcinoma. Cell.

[40] Guichard C, Amaddeo G, Imbeaud S, Ladeiro Y, Pelletier L, Maad IB (2012). Integrated analysis of somatic mutations and focal copy-number changes identifies key genes and pathways in hepatocellular carcinoma. Nat. Genet..

[41] Ho DWH, Chan LK, Chiu YT, Xu IMJ, Poon RTP, Cheung TT (2017). TSC1/2 mutations define a molecular subset of HCC with aggressive behaviour and treatment implication. Gut.

[42] Zender L, Spector MS, Xue W, Flemming P, Cordon-Cardo C, Silke J (2006). Identification and validation of oncogenes in liver cancer using an integrative oncogenomic approach. Cell.

[43] Akita H, Marquardt JU, Durkin ME, Kitade M, Seo D, Conner EA (2014). MYC activates stem-like cell potential in hepatocarcinoma by a p53-dependent mechanism. Cancer Res..

[44] Liu K, Lee J, Kim JY, Wang L, Tian Y, Chan ST (2017). Mitophagy controls the activities of tumor suppressor p53 to regulate hepatic cancer stem cells. Mol. Cell.

[45] Pandit H, Li Y, Li X, Zhang W, Li S, Martin RCG (2018). Enrichment of cancer stem cells via beta-catenin contributing to the tumorigenesis of hepatocellular carcinoma. BMC Cancer.

[46] Wang W, Li S, Liu P, Sideras K, van de Werken HJG, van der Heide M (2019). Oncogenic STRAP supports hepatocellular carcinoma growth by enhancing Wnt/beta-catenin signaling. Mol. Cancer Res.

[47] Chua HH, Tsuei DJ, Lee PH, Jeng YM, Lu J, Wu JF (2015). RBMY, a novel inhibitor of glycogen synthase kinase 3beta, increases tumor stemness and predicts poor prognosis of hepatocellular carcinoma. Hepatology.

[48] Kim H, Yoo JE, Cho JY, Oh BK, Yoon YS, Han HS (2013). Telomere length, TERT and shelterin complex proteins in hepatocellular carcinomas expressing “stemness”-related markers. J. Hepatol..

[49] Ng KY, Chai S, Tong M, Guan XY, Lin CH, Ching YP (2016). C-terminal truncated hepatitis B virus X protein promotes hepatocellular carcinogenesis through induction of cancer and stem cell-like properties. Oncotarget.

[50] Ching RHH, Sze KMF, Lau EYT, Chiu YT, Lee JMF, Ng IOL (2017). C-terminal truncated hepatitis B virus X protein regulates tumorigenicity, self-renewal and drug resistance via STAT3/Nanog signaling pathway. Oncotarget.

[51] Mani SK, Zhang H, Diab A, Pascuzzi PE, Lefrancois L, Fares N (2016). EpCAM-regulated intramembrane proteolysis induces a cancer stem cell-like gene signature in hepatitis B virus-infected hepatocytes. J. Hepatol..

[52] Ali N, Allam H, May R, Sureban SM, Bronze MS, Bader T (2011). Hepatitis C virus-induced cancer stem cell-like signatures in cell culture and murine tumor xenografts. J. Virol..

[53] Marquardt JU, Raggi C, Andersen JB, Seo D, Avital I, Geller D (2011). Human hepatic cancer stem cells are characterized by common stemness traits and diverse oncogenic pathways. Hepatology.

[54] Liu C, Liu L, Shan J, Shen J, Xu Y, Zhang Q (2013). Histone deacetylase 3 participates in self-renewal of liver cancer stem cells through histone modification. Cancer Lett..

[55] Ma S, Tang KH, Chan YP, Lee TK, Kwan PS, Castilho A (2010). miR-130b Promotes CD133(+) liver tumor-initiating cell growth and self-renewal via tumor protein 53-induced nuclear protein 1. Cell Stem Cell.

[56] Chai S, Ng KY, Tong M, Lau EY, Lee TK, Chan KW (2016). Octamer 4/microRNA-1246 signaling axis drives Wnt/beta-catenin activation in liver cancer stem cells. Hepatology.

[57] Chai S, Tong M, Ng KY, Kwan PS, Chan YP, Fung TM (2014). Regulatory role of miR-142-3p on the functional hepatic cancer stem cell marker CD133. Oncotarget.

[58] Li L, Tang J, Zhang B, Yang W, LiuGao M, Wang R (2015). Epigenetic modification of MiR-429 promotes liver tumour-initiating cell properties by targeting Rb binding protein 4. Gut.

[59] Wang X, Sun W, Shen W, Xia M, Chen C, Xiang D (2016). Long non-coding RNA DILC regulates liver cancer stem cells via IL-6/STAT3 axis. J. Hepatol..

[60] Wang Y, He L, Du Y, Zhu P, Huang G, Luo J (2015). The long noncoding RNA lncTCF7 promotes self-renewal of human liver cancer stem cells through activation of Wnt signaling. Cell Stem Cell.

[61] Zhu P, Wang Y, Wu J, Huang G, Liu B, Ye B (2016). LncBRM initiates YAP1 signalling activation to drive self-renewal of liver cancer stem cells. Nat. Commun..

[62] Zhu P, Wang Y, Huang G, Ye B, Liu B, Wu J (2016). lnc-beta-Catm elicits EZH2-dependent beta-catenin stabilization and sustains liver CSC self-renewal. Nat. Struct. Mol. Biol..

[63] Wu J, Zhu P, Lu T, Du Y, Wang Y, He L (2019). The long non-coding RNA LncHDAC2 drives the self-renewal of liver cancer stem cells via activation of Hedgehog signaling. J. Hepatol..

[64] Wang, Y., Zhu, P., Luo, J., Wang, J., Liu, Z., Wu, W. et al. LncRNA HAND2-AS1 promotes liver cancer stem cell self-renewal via BMP signaling. *EMBO J.* **38**, e101110 (2019). 10.15252/embj.2018101110 PMC6717889 31334575

[65] Zhu P, Wang Y, Du Y, He L, Huang G, Zhang G (2015). C8orf4 negatively regulates self-renewal of liver cancer stem cells via suppression of NOTCH2 signalling. Nat. Commun..

[66] Wan X, Cheng C, Shao Q, Lin Z, Lu S, Chen Y (2016). CD24 promotes HCC progression via triggering Notch-related EMT and modulation of tumor microenvironment. Tumour Biol..

[67] Hayashi H, Higashi T, Yokoyama N, Kaida T, Sakamoto K, Fukushima Y (2015). An Imbalance in TAZ and YAP expression in hepatocellular carcinoma confers cancer stem cell-like behaviors contributing to disease progression. Cancer Res..

[68] Xu L, Tong X, Zhang S, Yin F, Li X, Wei H (2016). ASPP2 suppresses stem cell-like characteristics and chemoresistance by inhibiting the Src/FAK/Snail axis in hepatocellular carcinoma. Tumour Biol..

[69] Lau EY, Lo J, Cheng BY, Ma MK, Lee JM, Ng JK (2016). Cancer-associated fibroblasts regulate tumor-initiating cell plasticity in hepatocellular carcinoma through c-Met/FRA1/HEY1 signaling. Cell Rep..

[70] Chan LH, Zhou L, Ng KY, Wong TL, Lee TK, Sharma R (2018). PRMT6 regulates RAS/RAF binding and MEK/ERK-mediated cancer stemness activities in hepatocellular carcinoma through CRAF methylation. Cell Rep..

[71] Fabregat I, Caballero-Diaz D (2018). Transforming growth factor-beta-induced cell plasticity in liver fibrosis and hepatocarcinogenesis. Front. Oncol..

[72] Wu J, Lu M, Li Y, Shang YK, Wang SJ, Meng Y (2016). Regulation of a TGF-beta1-CD147 self-sustaining network in the differentiation plasticity of hepatocellular carcinoma cells. Oncogene.

[73] Malfettone A, Soukupova J, Bertran E, Crosas-Molist E, Lastra R, Fernando J (2017). Transforming growth factor-beta-induced plasticity causes a migratory stemness phenotype in hepatocellular carcinoma. Cancer Lett..

[74] Almale, L., Garcia-Alvaro, M., Martinez-Palacian, A., Garcia-Bravo, M., Lazcanoiturburu, N., Addante, A. et al. c-Met signaling is essential for mouse adult liver progenitor cells expansion after transforming growth factor-beta-induced epithelial-mesenchymal transition and regulates cell phenotypic switch. *Stem Cells* **37**, 1108–1118 (2019). 10.1002/stem.3038 31108004

[75] Kim Y, Kang K, Lee SB, Seo D, Yoon S, Kim SJ (2019). Small molecule-mediated reprogramming of human hepatocytes into bipotent progenitor cells. J. Hepatol..

[76] Meindl-Beinker NM, Dooley S (2008). Transforming growth factor-beta and hepatocyte transdifferentiation in liver fibrogenesis. J. Gastroenterol. Hepatol..

[77] Roberts AB, Wakefield LM (2003). The two faces of transforming growth factor beta in carcinogenesis. Proc. Natl Acad. Sci. USA.

[78] Pardali K, Moustakas A (2007). Actions of TGF-beta as tumor suppressor and pro-metastatic factor in human cancer. Biochim Biophys. Acta.

[79] Tummala KS, Brandt M, Teijeiro A, Grana O, Schwabe RF, Perna C (2017). Hepatocellular carcinomas originate predominantly from hepatocytes and benign lesions from hepatic progenitor cells. Cell Rep..

[80] Cassim S, Raymond VA, Dehbidi-Assadzadeh L, Lapierre P, Bilodeau M (2018). Metabolic reprogramming enables hepatocarcinoma cells to efficiently adapt and survive to a nutrient-restricted microenvironment. Cell Cycle.

[81] Wei, Z., Jia, J., Heng, G., Xu, H., Shan, J., Wang, G. et al. Sirtuin-1/mitochondrial ribosomal protein S5 axis enhances the metabolic flexibility of liver cancer stem cells. *Hepatology* **70**, 1197–1213 (2019) 10.1002/hep.30622 30901096

[82] Ma, H. P., Chang, H. L., Bamodu, O. A., Yadav, V. K., Huang, T. Y., Wu, A. T. H. et al. Collagen 1A1 (COL1A1) is a reliable biomarker and putative therapeutic target for hepatocellular carcinogenesis and metastasis. *Cancers (Basel)* **11**, 786 (2019). 10.3390/cancers11060786 PMC6627889 31181620

[83] Tian, B., Luo, Q., Ju, Y. & Song, G. A soft matrix enhances the cancer stem cell phenotype of HCC cells. *Int. J. Mol. Sci*. **20**, 2831 (2019). 10.3390/ijms20112831 PMC6600428 31185668

[84] You Y, Zheng Q, Dong Y, Xie X, Wang Y, Wu S (2016). Matrix stiffness-mediated effects on stemness characteristics occurring in HCC cells. Oncotarget.

[85] Govaere O, Wouters J, Petz M, Vandewynckel YP, Van den Eynde K, Van, den Broeck A (2016). Laminin-332 sustains chemoresistance and quiescence as part of the human hepatic cancer stem cell niche. J. Hepatol..

[86] Cui CP, Wong CC, Kai AK, Ho DW, Lau EY, Tsui YM (2017). SENP1 promotes hypoxia-induced cancer stemness by HIF-1alpha deSUMOylation and SENP1/HIF-1alpha positive feedback loop. Gut.

[87] Liu K, Sun B, Zhao X, Wang X, Li Y, Qiu Z (2015). Hypoxia promotes vasculogenic mimicry formation by the Twist1-Bmi1 connection in hepatocellular carcinoma. Int. J. Mol. Med..

[88] Lai FB, Liu WT, Jing YY, Yu GF, Han ZP, Yang X (2016). Lipopolysaccharide supports maintaining the stemness of CD133(+) hepatoma cells through activation of the NF-kappaB/HIF-1alpha pathway. Cancer Lett..

[89] Pan QZ, Pan K, Wang QJ, Weng DS, Zhao JJ, Zheng HX (2015). Annexin A3 as a potential target for immunotherapy of liver cancer stem-like cells. Stem Cells.

[90] Li Y, Wang R, Xiong S, Wang X, Zhao Z, Bai S (2019). Cancer-associated fibroblasts promote the stemness of CD24(+) liver cells via paracrine signaling. J. Mol. Med. (Berl.).

[91] Zhao Z, Bai S, Wang R, Xiong S, Li Y, Wang X (2019). Cancer-associated fibroblasts endow stem-like qualities to liver cancer cells by modulating autophagy. Cancer Manag. Res..

[92] Jiang J, Ye F, Yang X, Zong C, Gao L, Yang Y (2017). Peri-tumor associated fibroblasts promote intrahepatic metastasis of hepatocellular carcinoma by recruiting cancer stem cells. Cancer Lett..

[93] Zhu F, Li X, Chen S, Zeng Q, Zhao Y, Luo F (2016). Tumor-associated macrophage or chemokine ligand CCL17 positively regulates the tumorigenesis of hepatocellular carcinoma. Med Oncol..

[94] Chen Y, Wen H, Zhou C, Su Q, Lin Y, Xie Y (2019). TNF-alpha derived from M2 tumor-associated macrophages promotes epithelial-mesenchymal transition and cancer stemness through the Wnt/beta-catenin pathway in SMMC-7721 hepatocellular carcinoma cells. Exp. Cell Res..

[95] Nakagawa H, Umemura A, Taniguchi K, Font-Burgada J, Dhar D, Ogata H (2014). ER stress cooperates with hypernutrition to trigger TNF-dependent spontaneous HCC development. Cancer Cell.

[96] Liu XL, Li FQ, Liu LX, Li B, Zhou ZPTNF-alpha (2013). HGF and macrophage in peritumoural liver tissue relate to major risk factors of HCC recurrence. Hepatogastroenterology.

[97] Muramatsu S, Tanaka S, Mogushi K, Adikrisna R, Aihara A, Ban D (2013). Visualization of stem cell features in human hepatocellular carcinoma reveals in vivo significance of tumor-host interaction and clinical course. Hepatology.

[98] Zhang Y, Zhao W, Li S, Lv M, Yang X, Li M (2019). CXCL11 promotes self-renewal and tumorigenicity of alpha2delta1(+) liver tumor-initiating cells through CXCR3/ERK1/2 signaling. Cancer Lett..

[99] He H, Wu J, Zang M, Wang W, Chang X, Chen X (2017). CCR6(+) B lymphocytes responding to tumor cell-derived CCL20 support hepatocellular carcinoma progression via enhancing angiogenesis. Am. J. Cancer Res..

[100] Wang XQ, Ongkeko WM, Chen L, Yang ZF, Lu P, Chen KK (2010). Octamer 4 (Oct4) mediates chemotherapeutic drug resistance in liver cancer cells through a potential Oct4-AKT-ATP-binding cassette G2 pathway. Hepatology.

[101] Cheung ST, Cheung PF, Cheng CK, Wong NC, Fan ST (2011). Granulin-epithelin precursor and ATP-dependent binding cassette (ABC)B5 regulate liver cancer cell chemoresistance. Gastroenterology.

[102] Chow EK, Fan LL, Chen X, Bishop JM (2012). Oncogene-specific formation of chemoresistant murine hepatic cancer stem cells. Hepatology.

[103] Park SY, Han J, Kim JB, Yang MG, Kim YJ, Lim HJ (2014). Interleukin-8 is related to poor chemotherapeutic response and tumourigenicity in hepatocellular carcinoma. Eur. J. Cancer.

[104] Zhou JJ, Deng XG, He XY, Zhou Y, Yu M, Gao WC (2014). Knockdown of NANOG enhances chemosensitivity of liver cancer cells to doxorubicin by reducing MDR1 expression. Int. J. Oncol..

[105] Zhang L, Ge C, Zhao F, Zhang Y, Wang X, Yao M (2016). NRBP2 overexpression increases the chemosensitivity of hepatocellular carcinoma cells via Akt signaling. Cancer Res..

[106] Yu G, Jing Y, Kou X, Ye F, Gao L, Fan Q (2013). Hepatic stellate cells secreted hepatocyte growth factor contributes to the chemoresistance of hepatocellular carcinoma. PLoS ONE.

[107] Lo J, Lau EY, Ching RH, Cheng BY, Ma MK, Ng IO (2015). Nuclear factor kappa B-mediated CD47 up-regulation promotes sorafenib resistance and its blockade synergizes the effect of sorafenib in hepatocellular carcinoma in mice. Hepatology.

[108] Lu S, Yao Y, Xu G, Zhou C, Zhang Y, Sun J (2018). CD24 regulates sorafenib resistance via activating autophagy in hepatocellular carcinoma. Cell Death Dis..

[109] Tong M, Fung TM, Luk ST, Ng KY, Lee TK, Lin CH (2015). ANXA3/JNK signaling promotes self-renewal and tumor growth, and its blockade provides a therapeutic target for hepatocellular carcinoma. Stem Cell Rep..

[110] Tong M, Che N, Zhou L, Luk ST, Kau PW, Chai S (2018). Efficacy of annexin A3 blockade in sensitizing hepatocellular carcinoma to sorafenib and regorafenib. J. Hepatol..

[111] Wang N, Wang S, Li MY, Hu BG, Liu LP, Yang SL (2018). Cancer stem cells in hepatocellular carcinoma: an overview and promising therapeutic strategies. Ther. Adv. Med. Oncol..

[112] Takai A, Dang H, Oishi N, Khatib S, Martin SP, Dominguez DA (2019). Genome-wide RNAi screen identifies PMPCB as a therapeutic vulnerability in EpCAM(+) hepatocellular carcinoma. Cancer Res..

[113] Li B, Cao Y, Meng G, Qian L, Xu T, Yan C (2019). Targeting glutaminase 1 attenuates stemness properties in hepatocellular carcinoma by increasing reactive oxygen species and suppressing Wnt/beta-catenin pathway. EBioMedicine.

[114] Nio K, Yamashita T, Okada H, Kondo M, Hayashi T, Hara Y (2015). Defeating EpCAM(+) liver cancer stem cells by targeting chromatin remodeling enzyme CHD4 in human hepatocellular carcinoma. J. Hepatol..

[115] Lai KKY, Kweon SM, Chi F, Hwang E, Kabe Y, Higashiyama R (2017). Stearoyl-CoA desaturase promotes liver fibrosis and tumor development in mice via a Wnt positive-signaling loop by stabilization of low-density lipoprotein-receptor-related proteins 5 and 6. Gastroenterology.

[116] Ma MKF, Lau EYT, Leung DHW, Lo J, Ho NPY, Cheng LKW (2017). Stearoyl-CoA desaturase regulates sorafenib resistance via modulation of ER stress-induced differentiation. J. Hepatol..

[117] Ma XL, Sun YF, Wang BL, Shen MN, Zhou Y, Chen JW (2019). Sphere-forming culture enriches liver cancer stem cells and reveals Stearoyl-CoA desaturase 1 as a potential therapeutic target. BMC Cancer.

[118] Tang KH, Ma S, Lee TK, Chan YP, Kwan PS, Tong CM (2012). CD133(+) liver tumor-initiating cells promote tumor angiogenesis, growth, and self-renewal through neurotensin/interleukin-8/CXCL1 signaling. Hepatology.

[119] Zhang L, Sun H, Zhao F, Lu P, Ge C, Li H (2012). BMP4 administration induces differentiation of CD133+ hepatic cancer stem cells, blocking their contributions to hepatocellular carcinoma. Cancer Res..

[120] Lee TK, Castilho A, Cheung VC, Tang KH, Ma S, Ng IO (2011). Lupeol targets liver tumor-initiating cells through phosphatase and tensin homolog modulation. Hepatology.

[121] Wu R, Murali R, Kabe Y, French SW, Chiang YM, Liu S (2018). Baicalein targets GTPase-mediated autophagy to eliminate liver tumor-initiating stem cell-like cells resistant to mTORC1 inhibition. Hepatology.

[122] Qin XY, Suzuki H, Honda M, Okada H, Kaneko S, Inoue I (2018). Prevention of hepatocellular carcinoma by targeting MYCN-positive liver cancer stem cells with acyclic retinoid. Proc. Natl Acad. Sci. USA.

[123] Zhong F, Cheng X, Sun S, Zhou J (2017). Transcriptional activation of PD-L1 by Sox2 contributes to the proliferation of hepatocellular carcinoma cells. Oncol. Rep..

[124] Chan LC, Li CW, Xia W, Hsu JM, Lee HH, Cha JH (2019). IL-6/JAK1 pathway drives PD-L1 Y112 phosphorylation to promote cancer immune evasion. J. Clin. Invest..

[125] Xu X, Xing B, Hu M, Xu Z, Xie Y, Dai G (2010). Recurrent hepatocellular carcinoma cells with stem cell-like properties: possible targets for immunotherapy. Cytotherapy.

[126] Rong XX, Wei F, Lin XL, Qin YJ, Chen L, Wang HY (2016). Recognition and killing of cancer stem-like cell population in hepatocellular carcinoma cells by cytokine-induced killer cells via NKG2d-ligands recognition. Oncoimmunology.

[127] Wang Y, Chen M, Wu Z, Tong C, Dai H, Guo Y (2018). CD133-directed CAR T cells for advanced metastasis malignancies: a phase I trial. Oncoimmunology.

